# A combinatorial oligogenic basis for the phenotypic plasticity between late-onset dilated and arrhythmogenic cardiomyopathy in a single family

**DOI:** 10.20517/jca.2021.15

**Published:** 2021-09-03

**Authors:** Kimia Pourebrahim, John Garrity Marian, Yanli Tan, Jeffrey T. Chang, Ali J. Marian

**Affiliations:** 1 Center for Cardiovascular Genetics, Institute of Molecular Medicine and Department of Medicine, University of Texas Health Sciences Center at Houston, Houston, TX 77030, USA.; 2 Upper school, St John’s school, Houston, TX 77030, USA.; 3 Integrative Biology & Pharm, University of Texas Health Sciences Center at Houston, Houston, TX 77030, USA

**Keywords:** Titin, plakophilin 2, desmoplakin, dilated cardiomyopathy, arrhythmogenic right ventricular cardiomyopathy, genetic, pathogenic variant, sudden death, mutation

## Abstract

**Introduction:**

Primary dilated cardiomyopathy (DCM) and arrhythmogenic right ventricular cardiomyopathy (ARVC) are the two common and distinct forms of hereditary cardiomyopathies caused by defined pathogenic variants (PVs) typically in different sets of genes. DCM is characterized by left ventricular dilatation, dysfunction, and failure, whereas ARVC classically involves the right ventricle and is characterized by fibrofatty infiltration of the myocardium. DCM is caused primarily by the PVs in genes encoding sarcomere and cytoskeletal protein, while ARVC is mainly a disease of the desmosome proteins. DCM and ARVC exhibit partial phenotypic and genetic overlaps.

**Aim:**

To analyze the genetic basis of the phenotypic heterogeneity of cardiomyopathy in members of a single family.

**Methods and Results:**

We recruited, clinically characterized, and performed whole-exome sequencing in five affected, three probably affected, and two clinically unaffected members of a single family. The family members mainly exhibited late-onset DCM associated with conduction defects and arrhythmias. One family member who died suddenly was diagnosed with the classic ARVC at autopsy and another presented with isolated ventricular tachycardia. A novel splicing (truncating) and a rare missense variant in the *TTN* gene, likely in cis, co-segregated with the phenotype in all affected and probably affected family members and were likely the causal variants. Several PVs and LPVs in other genes involved in cardiomyopathies and arrhythmias were also identified that seem to modify the expression of the phenotype. Notably, LPVs in the *DSP* and *PKP2* genes, which are known genes for ARVC, were identified in the family member who also carried the *TTN* variants but developed the classic ARVC.

**Conclusion:**

The findings indicate the causal role of the *TTN* variants, exhibiting an age-dependent penetrance in late-onset DCM, and highlight the potential modifying role of the concomitant LPVs in additional genes on the expression of the phenotype, including a phenotypic switch from the anticipated DCM to ARVC. The findings support an oligogenic basis of the cardiac phenotype in hereditary cardiomyopathies. A comprehensive genetic analysis involving all PVs and LPVs along with detailed phenotypic characterization is necessary to gain insights into the molecular pathogenesis of hereditary cardiomyopathies.

## INTRODUCTION

Hereditary cardiomyopathies are primary disorders of cardiac myocytes caused by defined mutations in genes encoding proteins involved in cardiac structure and function. The three most common forms of primary cardiomyopathies, classified according to their phenotypic characteristics, are hypertrophic cardiomyopathy (HCM), dilated cardiomyopathy (DCM), and arrhythmogenic cardiomyopathy (ACM). HCM and DCM represent the opposite ends of the phenotypic spectrum of the cardiac response to genetic mutations. The former is characterized by cardiac hypertrophy with a preserved or enhanced left ventricular ejection fraction (LVEF), and the latter manifests as a dilated heart with a thin wall and a reduced LVEF^[1,2]^. ACM is distinct from HCM and DCM, as cardiac arrhythmias are its cardinal manifestations, which occur early, prior to, and to some extent independent of cardiac dysfunction^[3]^. The classic form of ACM is referred to as arrhythmogenic right ventricular cardiomyopathy (ARVC), as it predominantly involves the right ventricle. The pathological hallmark of ARVC is fibro-fatty infiltration of the right ventricular myocardium^[4]^.

The genetic basis of hereditary cardiomyopathies has been partially elucidated and indicates considerable heterogeneity^[1–3]^. HCM is caused mainly by the mutations in genes that code for sarcomere proteins, such as myosin heavy chain 7, myosin binding protein C3, and cardiac troponin T (TNNT2) proteins (reviewed in^[1]^). DCM is mostly a disease of the sarcomere and cytoskeletal proteins (reviewed in^[2]^). Accordingly, truncating mutations in the *TTN* gene, encoding the giant protein titin, are responsible for about 20% of the DCM cases^[5]^. ACM is caused primarily by genes coding for the protein constituents of the desmosomes, such as *PKP2* and *DSP*, encoding plakophilin 2 and desmoplakin, respectively (reviewed in^[4]^).

There is a partial genetic overlap among hereditary cardiomyopathies, particularly between DCM and ACM. For example, the PVs in the *DSP* gene, encoding desmoplakin, are the classic causes of ACM but are also known to cause left-dominant ACM characterized by severe myocardial fibrosis^[6,7]^. Nevertheless, despite a partial genetic overlap, there is a distinct phenotypic clustering of each specific class of genes. Accordingly, PVs in genes encoding desmosome proteins mainly cause ACM, including ARVC, those in genes encoding cytoskeletal proteins and titin cause DCM, and those in genes encoding sarcomere proteins cause HCM.

A notable feature of the genetic basis of hereditary cardiomyopathies is incomplete penetrance, which is partly dependent on the presence of concomitant PVs and LPVs as well as environmental or external factors. The presence of the compound and multiple PVs or LPVs in genes implicated in cardiomyopathies raise the possible digenic and oligogenic basis of a subset of hereditary cardiomyopathies^[8]^. We describe a family whereby the DCM phenotype co-segregated with the inheritance of a truncating variant in the *TTN* gene (*TTN*tv) in several members, but the expression of the phenotype is affected by the presence of concomitant LPVs in other genes or external factors. Notably, a family member who also carried LPVs in the *PKP2* and *DSP* genes, known genes for ARVC, exhibited the classic ARVC, as opposed to the expected phenotype of DCM caused by the *TTN*tv.

## METHODS

### Regulatory approval and consent to participate

The human molecular genetic studies were approved by the institutional review board of the University of Texas Health Science Center at Houston (HSC-IMM-07–0016). The studies were in accord with the ethical principles described in the Declaration of Helsinki. The participants gave written informed consents prior to participation.

### Clinical studies

A pedigree was constructed, detailed medical history was obtained, and physical examination was performed on the participating family members. The participants underwent 12-lead electrocardiography (ECG) and complete echocardiography (M-mode, 2D, and Doppler). Selected family members underwent cardiac magnetic resonance (CMR) imaging per clinical indications, as judged by their care-providing physicians.

### Exome sequencing and variant annotation

Whole Exome Sequencing (WES) was performed in ten family members, including five phenotypically affected, two likely affected, and three phenotypically normal individuals on an Illumina platform. The sequence reads were aligned to the human reference genome GRCh37 (hg19). To identify the genetic variants in each exome, BAM files, containing the sequence alignment data, were preprocessed according to GATK best practices and analyzed as published with some modifications^[8,9]^. The variants were called by 4 different programs, namely GATK, Platypus, VarScan2, and FreeBayes, using the BETSY system to assess consistency among the variant callers and to weigh robustness of the detection^[10]^. The variants were initially filtered based on a minor allele frequency of < 5% in a European cohort from the 1000 Genomes Project as annotated by ANNOVAR, in accord with the ethnic background of the family^[11,12]^. Premature truncating variants, including stop gain, frameshift, and the canonical splice site variants, were considered PVs. Nonsynonymous variants in genes known to cause cardiomyopathies and/or arrhythmias with a CADD Phred (Combined Annotation-Dependent Depletion Phred) score of > 15 were considered LPVs and included for further analysis. SIFT, PolyPhen2, Mutation Taster, and other programs were also used to predict pathogenicity.

## RESULTS

### Clinical characteristics

**Figure 1.**
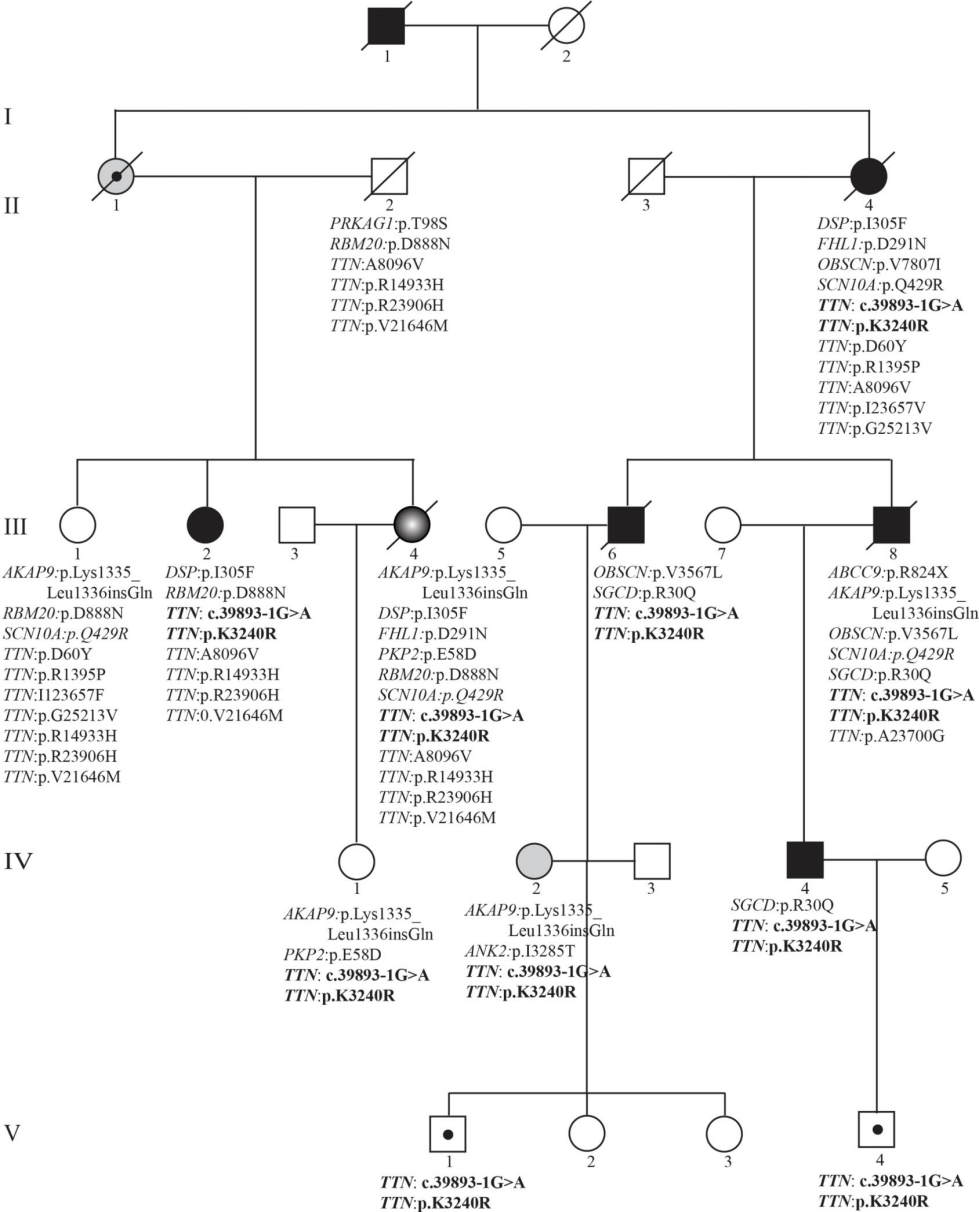
Pedigree and list of the likely pathogenic variants (LPVs) in genes known to cause cardiomyopathies. Generation and family member identifiers are listed, the latter under each symbol. Gene name and the amino acid change or splice variants are listed under each symbol, indicating presence of the variants in that exome. Square: male; Circle: female; Full circle and square: affected individuals; Open circle and square: unaffected individuals; slash through: deceased individuals. Circle and square with a dot at the center indicate carrier individuals or obligate carrier (II-1). Gray circle with white center identifies an individual with ARVC.

**Figure 2.**
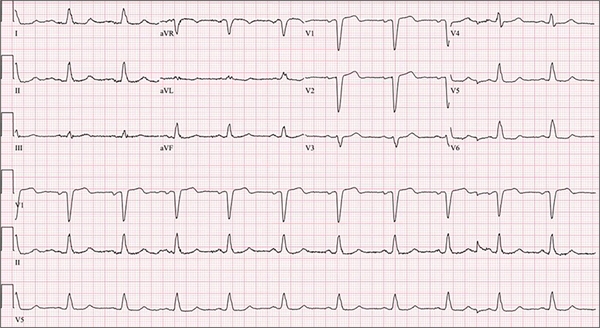
A 12-lead electrocardiogram in the proband (the upper three panels) and a rhythm strip (lower three panels). The electrocardiogram shows a normal sinus rhythm, probable left atrial enlargement, a normal PR interval, and an increased QRS duration with a normal axis and morphology, indicative of left bundle branch block.

**Figure 3.**
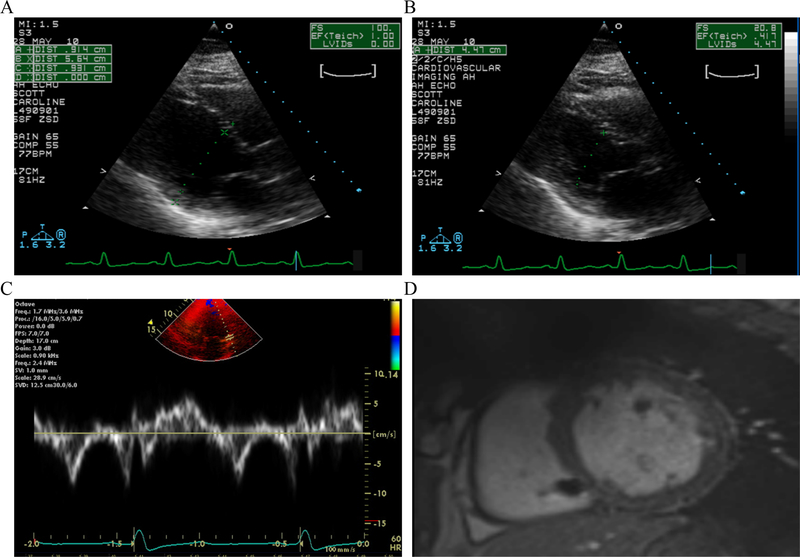
Echocardiogram and cardiac magnetic image resonance in the proband. Panels A and B show parasternal long axis view of the ventricles in diastole (panel A) and systole (Panel B). The left ventricular end diastolic and systolic diameters were measured 5.64 and 4.47 cm, respectively, and a calculated left ventricular fractional shortening of 21%. Panel C shows myocardial tissue Doppler velocities depicting low velocities. Panel D shows cross sectional myocardial images obtained by magnetic resonance imaging, which shows enlarged ventricles and absence of fibrofatty infiltration of the myocardium.

The clinical characteristics of five clinically affected and two likely clinically affected family members are presented in Table 1. The pedigree of the family is shown in Figure 1. Characteristics of specific family members are summarized as follows:

The proband (III-2) was a 65-year-old Caucasian female who presented several years earlier with episodes of palpitations and was found to have left bundle branch QRS morphology on a 12-lead ECG, very frequent ventricular premature beats, and short runs of nonsustained ventricular tachycardia (NSVT) on Holter monitoring [Figure 2]. She had no history of syncope, presyncope, or symptoms of heart failure. However, she had a strong family history of sudden cardiac death and heart failure. An echocardiogram and a CMR imaging showed a depressed and a normal right ventricular size and function. There was no evidence of fibrofatty infiltration of the myocardium on the CMR imaging. A high-resolution computed tomography of the chest showed no significant coronary calcification and an Agatston calcium score of zero. Over the course of subsequent years, echocardiograms showed a mildly dilated left ventricle (LV) with a reduced LVEF, as low as 30% [Figure 3A–C]. Likewise, a CMR showed LC dilatation and dysfunction and no evidence of fibro-adiposis [Figure 3D]. She was diagnosed to have primary DCM and underwent implantation of an internal cardioverter/defibrillator (ICD) and ventricular tachycardia ablation. She has remained stable without new symptoms, and her LV size and function have improved over time to normal values.

**Figure 4.**
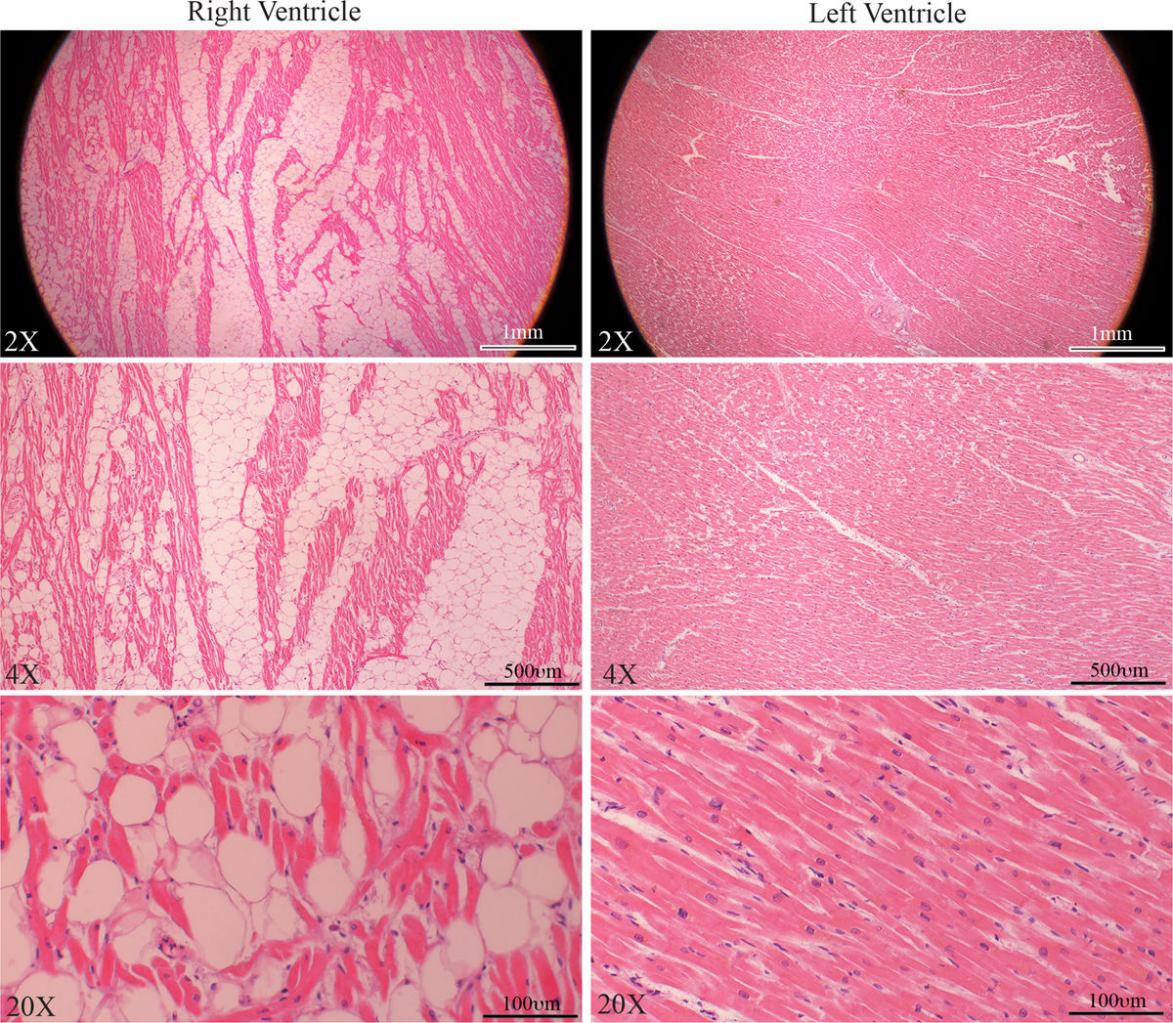
Histological findings in a family member with the classic ARVC. Masson trichrome stained myocardial sections, low (upper panel) and high magnification (lower panel) fields, from the right and left ventricles of family member III-4 who died suddenly. Sections from the right ventricle showing infiltration of the right ventricular wall with fibro-adipocytes, whereas the section from the left ventricle shows mild fibrosis.

Family member III-4 was a 54-year-old female who had a history of controlled systemic arterial hypertension. She presented a few years ago with chest pain and was found to have nonspecific T wave changes on 12-lead ECG and mild segmental hypokinesia in the septum and inferior apical walls during stress echocardiography. She had a normal right ventricular (RV) and LV size and function on multiple echocardiograms. She also underwent cardiac catheterization and coronary angiography, which did not show obstructive coronary lesions. A 24-h Holter monitor showed rare ventricular premature beats and no significant sustained or non-sustained supraventricular or ventricular arrhythmias, except for premature beats. She died suddenly at the age of 66 years. An autopsy showed four-chamber dilatation and cardiac hypertrophy with a heart weight of 496 grams and evidence of extensive fatty infiltration of the right ventricle. Microscopic examination showed fibro-fatty infiltration of the right ventricle, encompassing 50% to 80% of the right ventricular wall thickness [Figure 4]. The left ventricle was normal, except for microscopic evidence of mild hypertrophy and minimal fibrosis.

Three other affected family members (II-4, III-6, III-8) were diagnosed with DCM. All were initially symptomatic for heart failure at presentation and had significantly depressed LVEF (per verbal report in II-4). Three individuals had complete or incomplete left bundle branch block, and two had atrial fibrillation. Two had an ICD implanted because of syncope and/or frequent NSVT [Table 1].

Family member IV-2 presented with palpitations and presyncope and was found to have atrioventricular nodal reentrant tachycardia (AVNRT), NSVT, and PVCs. She was treated with antiarrhythmic medications and subsequently underwent radiofrequency ablation of AVNRT and an ICD placement. Echocardiography and CMR both showed normal right and left ventricular sizes and functions. There was no evidence of fibro-fatty infiltration of the myocardium on CMR.

One family member (IV-4) presented in his 40s and had a dilated LV with a reduced LVEF of 22% on an echocardiogram. He was a heavy alcohol drinker and was considered to have alcohol-related DCM. His LVEF was gradually recovered to the low 50s upon abstinence from alcohol.

The younger generation family members (generation V) were asymptomatic at the time of initial evaluation and had normal ECGs and echocardiograms.

### WES quality control metrics

Peripheral blood was collected after obtaining written consent; DNA was extracted and analyzed by short-read WES on an Illumina platform. The average number of total reads was ~45 million reads (range: 15 to 68 million reads), and the mean coverage was 33 folds (range: 9.3 to 66 folds). Detailed quality control metrics are presented in [Supplementary Table 1] and were considered in the analysis of the data in each exome.

Over > 800,000 variants that were identified across the 10 exomes. Approximately 140,000 variants had coverage of at least 20 reads, of which at least 5 reads contained the variants, compared to the reference genome. The list included ~30,500, ~21,000, and ~18,000 variants with a minor allele frequency of each variant of < 5%, < 1%, and 0.1%, respectively, in the 1000 Genomes Project dataset (1000g2015aug_eur). The latter database was used given the European ethnicity of the family. There were ~4400 non-synonymous single nucleotide variants (nsSNVs), 81 stop codon variants (10 start loss, 4 stop loss, and 67 stop gain), ~70 frameshift variants, 130 in-frame insertion, and ~730 splice region variants.

### Co-segregation of the variants with the phenotype

A total of 5000 putatively functional variants, comprised of nsSNVs, stop codon, frameshift, and splice region variants and a population frequency of < 5%, were analyzed for their co-segregation with the phenotype in five affected cases, two probably affected, and three unaffected family members [Figure 1]. Variants that were called at least by 2 different algorithms in three affected individuals with the best data quality (II-4, III-2, and III-4) were included. A total of 43 variants in 39 genes co-segregated with the affected status, not including those who were probably affected [Supplementary Table 2]. Among the candidate genes that contained co-segregating variants, *TTN* was the only gene that is expressed at high levels in cardiac myocytes, and given its well-established role in DCM, it had the strongest biological plausibility in causing the phenotype. The *ACOX3*, *DTX2*, *NBPF14* (NBPF26), *PDE4DIP*, and *TRIOBP* genes were expressed at low to medium, medium to medium to high levels in the myocardium, according to the Human Protein Atlas (https://www.proteinatlas.org/). Variants in this set of genes had low CADD Phred scores and were predicted to be benign or tolerated by several additional algorithms. Likewise, among the variants that co-segregated with the phenotype, those in the *CYP2D6*, *PARP4*, and *PJVK* had a CADD Phred score of > 15, but the corresponding genes were expressed at low levels in the heart.

A splicing acceptor PV (NM_133378:exon192:c.37112–1G > A) in the *TTN* gene, which is expected to lead to premature truncation of the TTN protein, co-segregated with the phenotype [Figure 1]. Likewise, a missense variant in the *TTN* gene, namely p.L3240R (NM_003319) or p.K3286R in exon 42 (NM_001267550), was predicted to be pathogenic or damaging by multiple algorithms (CADD Phred score of 18.05). The remaining variants resided in genes that are either not expressed in the heart or are expressed at low levels or are diffusely expressed in various tissues, and all had low *in silico* prediction of pathogenicity. All co-segregating variants and the assessment of their pathogenicity, including their expression in the heart, are listed in [Supplementary Table 2].

Both *TTN* variants were detected in 2 probably affected family members (IV-2 and IV-4). One member (IV2) presenting with cardiac arrhythmias, namely paroxysmal atrial tachycardia and NSVT carried the PVs in the *TTN* gene. She had a normal 12-lead ECG and normal right and left ventricular size and function on echocardiogram and CMR. She had no evidence of fibrofatty infiltration of the myocardium on the CMR. Thus, the phenotype was cardiac arrhythmias in the presence of normal cardiac structure and function. Individual IV-4 was clinically suspected of having alcohol-related DCM with severely depressed LVEF, who also carried the PVs in the *TTN* gene. The latter serving as the susceptibility variants, as the cardiac function significantly improved upon abstinence from alcohol consumption.

Individual IV-1 had inherited the *PKP2* and the *TTN* variants but not the *DSP* variant from her mother, who had ARVC. When last examined, she had right axis deviation on the ECG but had a normal right and left ventricular size and function on echocardiogram and CMR at age 36 years. She had no evidence of fibro-fatty infiltration of the myocardium on CMR.

There were four children in the family (V-1, V-2, V-3, and V-4) who were selectively screened for the *TTN* variants, and two were carriers. Their parents did not carry the *PKP2* or the *DSP* variants. The two *TTN* variant carriers had normal ECG and echocardiogram when last examined.

### Potential modifier variants in other genes known to cause cardiomyopathy and/or arrhythmias

Given the variability in the phenotypic expression of cardiomyopathy in the family members and the postmortem diagnosis of ARVC in one family member (III-4), the exome sequencing data were analyzed for the presence of potential modifier variants, defined as those with a MAF < 0.05 and a CADD Phred score of 15 or higher in 97 genes that have been associated with hereditary cardiomyopathies or cardiac arrhythmias [Supplementary Table 3]. Such variants were identified in 7 genes, including the *TTN* gene, in the affected family members [Table 2]. None of these variants, except for the *TTN* variants, co-segregated with the phenotype. However, they were notable for their potential impacts on the phenotype, including the presence of two heterozygous PVs and LPVs in the *DSP* and the *PKP2* genes, known genes for ARVC, in the family member who exhibited with the classic ARVC at autopsy [Figure 1, Figure 4, and Table 2]. This individual had the highest load of PVs and LPVs, involving 7 different genes (5 genes involved in cardiomyopathy and 2 in cardiac arrhythmias). The *DSP* variant was also present in two additional affected family members who also have the *TTN* variants and presented with classic DCM. Other notable variants were in the *FHL1*, *OBSCN*, *PRKAG1*, *RBM20*, and *SGCD* genes, which are implicated in hereditary cardiomyopathies^[13–15]^.

Given the preponderance of conduction defects and cardiac arrhythmias in the family members, the exome sequencing data were also analyzed for the presence of uncommon PVs and LPVs, and those with a CADD Phred score of > 15. The results, shown in Figure 1 and Table 3, are notable for the presence of 5 notable variants, in addition to those also involved in cardiomyopathies, in the *ABCC9*, *AKAP9*, *ANK2*, and *SCN10A* in the family members. Individual III-4, who died suddenly despite a normal cardiac function, carried LPVs in the *AKAP9* and *SCN10A* genes, both implicated in cardiac arrhythmias^[16,17]^.

## DISCUSSION

The data indicate the pathogenic role of the splicing variant (c.18197–1G > A) in the *TTN* gene, which is expected to lead to premature truncation of the TTN protein, in late-onset DCM, as the variant co-segregates with an inheritance of the phenotype in the family members. A missense mutation (NM_003319, c.A9719G, p.K3240R) also co-segregate with DCM in the family member and is likely in cis with the splicing variant. The data also suggest contributions of multiple LPVs in genes known to cause cardiomyopathies and cardiac arrhythmias to the heterogeneity of the phenotype in the family members. A notable example was the concomitant presence of LPVs in the *PKP2* and *DSP* genes, known to cause ARVC, in addition to the *TTN* variants in a family member who was found to have the classic ARVC at autopsy^[18–20]^. The family member had PVs and LPVs in 7 genes implicated in hereditary cardiomyopathies and/or cardiac arrhythmias, including LPVs in *SCN10A* and *AKAP9*, which are implicated in cardiac arrhythmias^[16,17]^. Overall, the data imply contributions of several PVs and LPVs to the expression of a heterogenous group of phenotypes observed in the affected members of a single family.

The data also illustrate inadequate sensitivity of the ECG and echocardiography in the diagnosis of ARVC, particularly when only the RV is involved. Notably, the family member with the ARVC had multiple echocardiograms and ECGs, which were considered unremarkable without evidence of right or left ventricular enlargement or dysfunction. Nonetheless, at autopsy, she was found to have chamber enlargement and extensive infiltration of the right ventricular myocardium with adipocytes [Figure 2].

The data also suggest incomplete penetrance as well as phenotypic heterogeneity of the PVs in the *TTN* gene, along with the undulating nature of the phenotype in the family members. Accordingly, one family member who carried the *TTN*tv showed no discernible phenotype in her 30s. The second exhibited supraventricular and ventricular arrhythmias, requiring ablation and an ICD placement, and had normal RV and LV size and function. The third family member with the *TTN*tv presented with severe heart failure and LV dysfunction, which partially recovered upon abstinence from alcohol consumption. In the latter family member, *TTN*tv seemingly functioned as a susceptibility allele leading to unmasking or precipitation of DCM upon alcohol consumption. Finally, a fourth family member, who had LV enlargement with depressed systolic function as well as NSVT, had a near-normal cardiac size and function upon medical therapy several years after the initial diagnosis. Two younger members of the family had inherited the *TTN* variants and showed no phenotype in the second decade of life. The findings, overall, suggest an age-dependent penetrance of the *TTN* variants, typically presenting in the 6th decade of life, whereby expression of the phenotype is partially conditional to the presence of concomitant LPVs in other genes associated with cardiomyopathies and cardiac arrhythmias or the presence of external factors, such as alcohol consumption. These findings illustrate the complexity of genotype-phenotype correlation in hereditary cardiomyopathies and advocate for an oligogenic basis of the phenotype and the need for a comprehensive genetic analysis to identify all PVs and LPVs in each genome in the clinical genetic testing^[21]^.

A notable feature of the phenotype in the family members, in addition to DCM, was the preponderance of conduction defect, namely left bundle branch block (LBBB), atrial fibrillation, and ventricular arrhythmias. Although the PVs in the *TTN* gene have been associated with atrial fibrillation and ventricular arrhythmias, the role of the *TTN* gene in intraventricular conduction defect, namely LBBB, is unclear^[22,23]^. Screening of the exome data for the PV and LPVs led to the identification of LPVs in four genes known to be associated with cardiac arrhythmias and conduction defects in several family members. PVs and LPVs in the ABCC9, *AKAP9*, *ANK2*, and *SCN10A* have been implicated in conduction defects and arrhythmias, particularly the long QT syndromes and conduction defects^[16,17,24,25]^. None of the PVs and LPVs segregated with the phenotype in the family members. Overall, the contributions of these variants to the pathogenesis of cardiac conduction defects and arrhythmias in members of the family, while plausible, could be not ascertained conclusively.

Despite the incomplete penetrance and phenotypic heterogeneity, evidence for the causal role of the *TTN*tv in the pathogenesis of DCM is strong and supported by the co-segregation analysis in the family members. The *TTN* gene is also implicated as a cause of ARVC, albeit the data are scant^[20]^. The modifier roles of the PVs or LPVs in the *PKP2*, *DSP*, *SCN10A*, and others identified in the family members benefit from biological plausibility and the well-established roles of these genes in cardiomyopathies and/or arrhythmias^[17,18,19]^. Nevertheless, the observations are also in accord with the notion that the ensuing phenotype, even in single-gene disorders, is influenced by a number of genetic and non-genetic factors, exerting a gradient of phenotypic effects with the final phenotype being the outcome of stochastic, multilayer, and nonlinear interactions among numerous genetic and non-genetic determinants^[21,26,27]^. Despite the plausibility, the role of the PVs and LPVs as the modifiers of the phenotype caused by the PVs in *TTN* gene in the family members could not be discerned unambiguously. The concern, by and large, is applicable to discerning the modifying roles of the genetic variants in single-gene disorders because of the plethora of the genetic variants in each genome and the relatively modest effect sizes of the variants, which necessitate comprehensive approaches either through family studies or genome-wide case-control association studies^[21]^. Thus, the findings regarding the modifying effects of the variants are provisional in nature, requiring replication in additional studies and testing through experimentation.

In conclusion, the findings indicate the pathogenic role of the *TTN* variants in late-onset familial DCM associated with cardiac conduction defects and arrhythmias. In addition, the findings suggest modifying effects of several PVs and LPVs in genes involved in cardiomyopathies and arrhythmias on the heterogeneity of phenotype expression, including phenotypic plasticity leading to a switch from the anticipated DCM to that of the classic ARVC. The findings point to an oligogenic basis of the cardiac phenotype in hereditary cardiomyopathies and emphasize the necessity of a comprehensive approach to analyzing all PV and LPVs in each exome in order to gain insight into the genetic and phenotypic spectrums of hereditary cardiomyopathies and arrhythmias.

## Supplementary Material

Supplementary Material

## Figures and Tables

**Table 1.** Clinical characteristics of the family members

			Affected			Probably affected	

	II-4	III-2	III-4	III-6	III-8	IV-2	IV-4
Age (years)	80	57	67	56	54	42	45
Sex (M/F)	F	F	F	M	M	F	M
Weight (Kg)	82	111	92	95	82	64.5	83
Height (cm)	155	165	157	173	173	165	180
BMI (Kg/m^2^)	34	40.8	37.3	31.7	27.4	23.7	25.6
Presentation	Heart failure	Palpitations	SCD	Heart failure	Syncope, heart failure	Palpitations, AVNRT, NSVT	Heart failure
**ECG and Holter monitoring**							
Rhythm	Atrial fibrillation	Sinus rhythm	Sinus rhythm	Atrial fibrillation ablation, paced rhythm	Sinus bradycardia	Sinus rhythm	Sinus rhythm
Conduction defect	LBBB	LBBB	None	IVCD	LBBB	None	None
Arrhythmias	Atrial fibrillation, ICD implantation	NSVT, VT ablation, ICD implantation	PVCs	PACs and PVCs, ICD implantation	VT, ICD implantation	PVCs, NSVT, ICD implantation	PVCs
Others	Low voltage QRS, ST & T changes changes	LAE, ST & T changes	LAE, ST & T changes	Lateral Q waves	None	Low voltage QRS	None
**Echocardiographic findings**							
IVST (cm)	NA	0.8	1.1	1.0	1.0	0.7	0.7
LVEDD (cm)	NA	5.3	5.6	6.2	6.8	5.1	6.5
LVESD (cm)	NA	4.1	3.8	5.6	4.5	3.5	5.7
LVEF (%)	NA	40	65	< 20	30	58	25
RV size and function	NA	Normal	RVIDd: 2.3 Normal	Normal	Normal	Normal	Dilated and hypokinetic
Other findings	NA	LAE	Mild MR and AI	LAE, PA pressure: 56 mmHg	LAE, mild MR	TAPSE: 26 mm	PA pressure: 58 mmHg
**CMR findings**							
LVEDD (cm)	NA	5.1	NA	NA	NA	Normal size	NA
LVESD (cm)	NA	4.1	NA	NA	NA	Normal size	NA
LVEF (%)	NA	52	NA	NA	NA	58.5	NA
RVEF (%)	NA	57	NA	NA	NA	53.7	NA
Others	NA	No fibro-adiposis	Fibro-adiposis involving 50% to 80% of the RV wall thickness. No LV involvement	NA	NA	No evidence of fibro-adiposis	NA
Other diagnostic tests & interventions	Reduced LVEF on echocardiograms (verbal report)	Coronary calcium score: 0 VT ablation	No obstructive coronary lesion	No obstructive coronary lesion	No obstructive coronary lesion	RF ablation of ANVRT	Normal myocardial perfusion tomography No obstructive coronary lesion

M/F: Male/female; BMI: body mass index; SCD: sudden cardiac death; AVNRT: atrioventricular nodal reentrant tachycardia; NSVT: non-sustained ventricular tachycardia; LBBB: left bundle branch block; IVCD: intraventricular conduction defect; ICD: internal cardioverter defibrillator; VT: ventricular tachycardia; PACs: premature atrial contraction; PVCs: premature ventricular contractions; LAE: left atrial enlargement; IVST: interventricular septal thickness; LVEDD: left ventricular end diastolic diameter; LVESD: left ventricular end systolic diameter; LVEF: left ventricular ejection fraction; RV: right ventricle; RVID: right ventricular internal diameter; NA: not available; MR: mitral regurgitation; TAPSE: tricuspid annular plane systolic excursion; PA: pulmonary artery; RVEF: right ventricular ejection fraction; LV: left ventricle; RF: radiofrequency.

**Table 2.** Uncommon variants in genes known to cause cardiomyopathies in the family members

Gene	NCBI Ref Seq	Coding Sequence change	Amino acid sequence change	MAF; 1000g-Eur	CADD Phred score	Affectied					Probably affected		Unaffected		
						II-4	III-2	III-4	III-6	III-8	IV-2	IV-4	II-2	III-1	IV-1

*DSP*	NM_001008844	c.A913T	p.I305F	0.041	27.3	+	+	+	−	−	−	−	−	−	−
*FHL1*	NM_001159699	c.G871A	p.D291N	0.018	15.2	+	−	+	−	−	−	−	−	−	−
	NM_001159701	c.G910A	p.D304N												
	NM_001159704	c.G823A	p.D275N												
*OBSCN*	NM_001098623	c.G10699C	p.V3567L	0.002	35.0	−	−	−	+	−	−	−	−	−	−
	NM_001271223	c.G11986C	p.V3996L												
	NM_001098623	c.G23419A	p.V7807I	0.022	20.1	+	−	−	−	+	−	−	−	−	−
	NM_001271223	c.G26290A	p.V8764I												
*PKP2*	NM_001005242	c.G174T	p.E58D	0.012	16.9	−	−	+	−	−	−	−	−	−	+
	NM_004572														
*PRKAG1*	NM_001206709	c.C293G	p.T98S	0.024	27.9	−	−	−	−	−	−	−	+	−	−
	NM_001206710	c.C170G	p.T57S												
	NM_002733	c.C266G	p.T89S												
*RBM20*	NM_001134363	c.G2662A	p.D888N	0.004	21.6	−	+	+	−	−	−	−	+	+	−
*SGCD*	NM_001128209	c.G89A	p.R30Q	ND	33.0	−	−	−	+	+	−	+	−	−	−
	NM_000337	c.G92A	p.R31Q												
*TTN*	**NM_003319**	**c.A9719G**	**p.K3240R**	**0.001**	**18.1**	**+**	**+**	**+**	**+**	**+**	**+**	**+**	**−**	**−**	**+**
	**NM_001256850**	**c.A9857G**	**p.K3286R**												
	**NM_133437**	**c.18197–1G>A**	**Splicing**	**ND**	**26.9**	**+**	**+**	**+**	**+**	**+**	**+**	**+**	**−**	**−**	**+**
	**NM_001256850**	**c.39893–1G>A**													
	**NM_003319**	**c.17621–1G>A**													
	**NM_133378**	**c.37112–1G>A**													
	**NM_001267550**	**c.44816–1G>A**													
	**NM_133432**	**c.17996–1G>A**													
	NM_003319	c.G71717A	p.R23906H	0.048	23.5	−	+	+	−	−	−	−	+	+	−
	NM_133432	c.G72092A	p.R24031H												
	NM_133437	c.G72293A	p.R24098H												
	NM_133378	c.G91208A	p.R30403H												
	NM_001256850	c.G93989A	p.R31330H												
	NM_001267550	c.G98912A	p.R32971H												
	NM_003319	c.C71099G	p.A23700G	0.002	22.5	−	−	−	−	+	−	−	−	−	−
	NM_133432	c.C71474G	p.A23825G												
	NM_133437	c.C71675G	p.A23892G												
	NM_133378	c.C90590G	p.A30197G												
	NM_001256850	c.C93371G	p.A31124G												
	NM_001267550	c.C98294G	p.A32765G												
	NM_003319	c.G4184C	p.R1395P	0.012	16.7	+	−	−	−	−	−	−	−	+	−
	NM_001256850	c.G4322C	p.R1441P												
	NM_001256850	c.G178T	p.D60Y	0.009	15.7	+	−	−	−	−	−	−	−	+	−
	NM_003319	c.C24287T	p.A8096V	0.021	16.6	+	+	+	−	−	−	−	+	−	−
	NM_133432	c.C24662T	p.A8221V												
	NM_133437	c.C24863T	p.A8288V												
	NM_133378	c.C43778T	p.A14593V												
	NM_001256850	c.C46559T	p.A15520V												
	NM_001267550	c.C51482T	p.A17161V												
	NM_003319	c.A70969T	p.I23657F	0.023	16.6	+	−	−	−	−	−	−	−	+	−
	NM_133432	c.A71344T	p.I23782F												
	NM_133437	c.A71545T	p.I23849F												
	NM_133378	c.A90460T	p.I30154F												
	NM_001256850	c.A93241T	p.I31081F												
	NM_001267550	c.A98164T	p.I32722F												
	NM_003319	c.G75638T	p.G25213V	0.032	15.1	+	−	−	−	−	−	−	−	+	−
	NM_133432	c.G76013T	p.G25338V												
	NM_133437	c.G76214T	p.G25405V												
	NM_133378	c.G95129T	p.G31710V												
	NM_001256850	c.G97910T	p.G32637V												
	NM_001267550	c.G102833T	p.G34278V												
	NM_003319	c.G44798A	p.R14933H	0.047	15.0	−	+	+	−	−	−	−	+	+	−
	NM_133432	c.G45173A	p.R15058H												
	NM_133437	c.G45374A	p.R15125H												
	NM_133378	c.G64289A	p.R21430H												
	NM_001256850	c.G67070A	p.R22357H												
	NM_001267550	c.G71993A	p.R23998H												
	NM_003319	c.G64936A	p.V21646M												
	NM_133432	c.G65311A	p.V21771M	0.049	15.0	−	+	+	−	−	−	−	+	+	−
	NM_133437	c.G65512A	p.V21838M												
	NM_133378	c.G84427A	p.V28143M												
	NM_001256850	c.G87208A	p.V29070M												
	NM_001267550	c.G92131A	p.V30711M												

Gene symbols are per HUGO nomenclature. RefSeq: Reference sequence; MAF: Minor allele frequency; 1000g-Eur: 1000 European Genomes data; CADD: Combined annotation dependent depletion; ND: not detected. Variants co-segregating with the phenotype are shown in bold.

**Table 3.** Uncommon variants in genes known to cause cardiac arrhythmias in the family members

Gene	NCBI Ref Seq	Coding Sequence change	Amino acid sequence change	MAF; 1000g-Eur	CADD Phred score	Affectied					Probably affected		Unaffected		
						II-4	III-2	III-4	III-6	III-8	IV-2	IV-4	II-2	III-1	IV-1

*ABCC9*	NM_005691	c.C2470T	p.R824X	ND	39.0	−	−	−	−	+	−	−	−	−	−
*AKAP9*	ENSG00000127914	c.4004_4006dupAAC	p.Lys1335_Leu1336insGln	ND	NA	−	−	+	−	+	+	−	−	+	+
*ANK2*	NM_001148	c.T9854C	p.I3285T	0.008	16.1	−	−	−	−	−	+	−	−	−	−
*SCN10A*	NM_001293306	c.A1286G	p.Q429R	ND	23.4	+	−	+	−	+	−	−	−	+	−

Gene symbols are per HUGO nomenclature. RefSeq: Reference sequence; MAF: Minor allele frequency; 1000g-Eur: 1000 European Genomes data; CADD: combined annotation dependent depletion; ND: not detected. Variants co-segregating with the phenotype are shown in bold.
