# Supplementary material for: A combinatorial oligogenic basis for the phenotypic plasticity between late-onset dilated and arrhythmogenic cardiomyopathy in a single family
Source: J Cardiovasc Aging. Author manuscript; Available in PMC 2021 Nov 16. (PMC8594872; doi:10.20517/jca.2021.15)
Supplement: Supplementary Material [file NIHMS1739287-supplement-Supplementary_Material.pdf]

Online Table I: Whole Exome Sequencing Data Quality Metrics

Sample	Total Reads	Mean Read Length	Mean Coverage	% Reads Aligned	% Duplicated	% Off Bait	% Usable Bases on Target
II-2	32,711,945	71.9	13.4	98.29	10.94	32.64	36.17
II-4	67,588,605	138.7	64.9	98.87	8.68	7.84	43.98
III-1	38,620,282	72.4	21.5	98.75	15.54	10.69	48.83
III-2	58,760,922	136.3	56.9	98.60	8.03	10.38	45.16
III-4	66,784,500	138.5	65.6	98.82	7.58	7.86	45.06
III-6	14,944,403	72.9	9.3	99.20	10.52	10.71	54.05
III-8	40,723,737	72.6	23.4	98.98	15.53	10.46	50.31
IV-2	34,902,483	73.0	20.4	99.23	15.96	10.67	50.96
IV-4	37,296,478	73.0	21.7	99.30	16.26	10.64	50.60
IV-1	59,281,944	73.0	32.4	99.23	20.34	11.06	47.58

Online Table 2<br>Genetic Variants Co-segregating with the Phenotype Family 039						
**Gene**	**Variant**			**MAF (1,000G,Eur)**	**CADD-Phred score**	**Expression in cardiac myocytes**
	**Ref Seq**	**Coding sequence**	**AA change**			
**Biologically Plausible**						
TTN	NM_003319	c.A9719G	p.K3240R	0.001	18.05	High
	NM_133437	c.18197-1G>A	Splice acceptor	ND	26.9	
**Biologically implausible**						
ACOX3	NM_001101667	c.A448C	p.M150L	0.010	3.06	Low to Medium
CCDC74A	NM_001258305	c.G688A	p.G230S	ND	0.01	Low
CYP2D6	NM_001025161	c.G941A	p.R314H	ND	34.0	Low
		c.G833T	p.R278L	ND	14.89	
DNAH7	NM_018897	c.G2637T	p.M879I	0.035	3.92	Low
DTX2	NM_001102595	c.C1093T	p.R365C	ND	13.4	Medium
FRG2B	NM_001080998	c.G25A	p.D9N	ND	10.46	Low
FSIP2	NM_173651	c.T17381C	p.M5794T	ND	13.82	Low
GOLGA8A	NM_181077	c.G200C	p.R67P	ND	4.46	Low
GORASP2	NM_001201428	c.C1091T	p.S364F	ND	11.62	Low
HLA-DRB1	NM_002124	c.T397G	p.S133A	ND	0	Low
IL32	NM_001012636	c.A488G	p.D163G	ND	0.81	Low
	ENSG00000008517	c.454dupG	p.Asp152fs	ND	NA	
KRTAP10-7	NM_198689	c.A475G	p.I159V	ND	0.01	Low
LILRA6; LILRB3	NM_001081450	c.T206G	p.L69W	ND	0.01	Low
MUC6	NM_005961	c.C5494T	p.P1832S	ND	11.67	Low
MUC12	NM_001164462	c.G58C	p.V20L	ND	0.13	Low
NBPF14; NBPF26	NM_001351372	c.A592G	p.K198E	ND	0.01	Medium
OR11H12	NM_001013354	c.G596T	p.R199L	ND	0.0	Low
OR2T33	NM_001004695	c.T479C	p.V160A	ND	0.01	Low
		c.T590G	p.M197R	ND	6.23	
		c.T145C	p.W49R	ND	0.05	
PARP4	NM_006437	c.T3116C	p.I1039T	ND	17.08	Low
PDE4DIP	NM_001350520	c.G7358A	p.R2453Q	ND	0.01	Medium to High
	NM_001198832	c.C211A	p.L71I	ND	19.2	
PIK3C2G	NM_004570	c.T3869G	p.V1290G	0.022	0.03	Low
PJVK	NM_001353776	c.G880A	p.G294R	0.025	16.89	Low
POM121	NM_172020	c.G2254A	p.V752I	ND	0.01	Low
		c.T2266C	p.Y756H	ND	0.06	
PRAMEF1	NM_001294139	c.A423C	p.R141S	ND	5.87	Low

PRB1	NM_001367912	c.G1249A	p.E417K	ND	2.98	Low
RFPL3	NM_001098535	c.C838T	p.R280C	ND	10.28	Low
RGPD5; RGPD8	NM_001164463	c.G4708A	p.G1570R	ND	8.95	Low
RP1L1	NM_178857	c.G3955A	p.A1319T	ND	2.93	Low
TPSD1	NM_012217	c.G274A	p.A92T	ND	0.60	Low
TRIOBP	NM_001039141	c.G1478A	p.S493N	ND	4.19	Medium
TUBA3D	NM_080386	c.C661A	p.R221S	ND	3.46	Low
UBXN11	NM_001077262	c.G1126T	p.G376C	ND	8.13	Low
VCX3A	NM_016379	c.G556A	p.V186M	ND	10.55	Low
ZNF730	NM_001277403	c.C1235T	p.T412I	0.040	6.31	Low
SERHL2	ENSG00000182841	n.588+2dupT	Splice donor-intron	ND	NA	Low

Abbreviations: Gene symbols are per HUGO nomenclature, Ref Seq: Reference sequence; CADD: Combined Annotation Dependent Depletion; AA: amino acid; NA: Not applicable; ND: Not detected. 1000G,Eur: The 1,000 genomes project in the European population.

TTN gene and its variants are listed in bold letter, because of high likelihood pathogenicity.

Online Table 3

List of Genes Associated with Cardiomyopathies and/or Arrhythmias

Gene Symbol	Locus
ABCC9	(12p12.1)
ACTC1	(15q14)
ACTN2	(1q43)
AKAP9	(7q21.2)
ANK2	(4q25-26)
ANKRD1	(10q23.31)
ASPH	(8q12.3)
BAG3	(10q26.11)
CACNA1C	(12p13.33)
CACNA1D	(3p21.1)
CACNA2D1	(7q21.11)
CACNB2	(10p12.33-12.31)
CALM1	(14q32.11)
CALM2	(2p21)
CALM3	(19q13.32)
CASQ2	(1p13.1)
CAV3	(3p25.3)
CAVIN4	(9q31.1)
CHRM2	(7q33)
CRYAB	(11q23.1)
CSRP3	(11p15.1)
DES	(2q35)
DMD	(Xp21.2-21.1)
DOLK	(9q34.11)
DPP6	(7q36.2)
DSC2	(18q12.1)
DSG2	(18q12.1)
DSP	(6p24.3)
DTNA	(18q12.1)
EMD	(Xq28)
FHL1	(Xq27.2)
FHL2	(2q12.2)

GATAD1	(7q21.2)
GJA5	(1q21.2)
GLA	(Xq22.1)
GPD1L	(3p22.3)
HCN4	(15q24.1)
ILK	(11p15.4)
JPH2	(20q13.12)
JUP	(17q21.2)
KCNA5	(12p13.32)
KCND3	(1p13.2)
KCNE1	(21q22.12)
KCNE2	(21q22.11)
KCNE3	(11q13.4)
KCNE5	(Xq23)
KCNH2	(7q36.1)
KCNJ2	(17q24.3)
KCNJ5	(11q24.3)
KCNJ8	(12p12.1)
KCNQ1	(11p15.5-15.4)
LAMA4	(6q21)
LAMP2	(Xq24)
LDB3	(10q23.2)
LMNA	(1q22)
MYBPC3	(11p11.2)
MYH6	(14q11.2)
MYH7	(14q11.2)
MYL2	(12q24.11)
MYL3	(3p21.31)
MYLK2	(20q11.21)
MYOZ2	(4q26)
MYPN	(10q21.3)
NEBL	(10p12.31)
NEXN	(1p31.1)
NPPA	(1p36.22)
PDLIM3	(4q35.1)
PKP2	(12p11.21)
PLN	(6q22.31)

PRDM16	(1p36.32)
PRKAG1	(7q36.1)
PTPN11	(12q24.13)
RAF1	(3p25.2)
RANGRF	(17p13.1)
RBM20	(10q25.2)
RYR2	(1q43)
SCN10A	(3p22.2)
SCN1B	(19q13.11)
SCN2B	(11q23.3)
SCN3B	(11q24.1)
SCN4B	(11q23.3)
SCN5A	(3p22.2)
SGCD	(5q33.2-33.3)
SLMAP	(3p14.3)
SNTA1	(20q11.21)
TAFAZZIN	(Xq28)
TCAP	(17q12)
TMEM43	(3p25.1)
TNNC1	(3p21.1)
TNNI3	(19q13.42)
TNNT2	(1q32.1)
TPM1	(15q22.2)
TRDN	(6q22.31)
TRPM4	(19q13.33)
TTN	(2q31.2)
TTR	(18q12.1)
VCL	(10q22.2)
